# An Efficient, Short Stimulus PANC-1 Cancer Cell Ablation and Electrothermal Therapy Driven by Hydrophobic Interactions

**DOI:** 10.3390/pharmaceutics15010106

**Published:** 2022-12-28

**Authors:** Maria P. Meivita, Denise Lee, J Shamita Naikar, Shao-Xiang Go, Wey Chyi Teoh, Yaw Sing Tan, Natasa Bajalovic, Desmond K. Loke

**Affiliations:** 1 Department of Science, Mathematics and Technology, Singapore University of Technology and Design, Singapore 487372, Singapore; 2 Office of Innovation, Changi General Hospital, Singapore 529889, Singapore; 3 Department of Radiology, Changi General Hospital, Singapore 529889, Singapore; 4 Bioinformatics Institute, Agency for Science, Technology and Research (A*STAR), Singapore 138671, Singapore

**Keywords:** ablation, cancer cell, phage, MoS_2_, molecular dynamics, agent

## Abstract

Promising results in clinical studies have been demonstrated by the utilization of electrothermal agents (ETAs) in cancer therapy. However, a difficulty arises from the balance between facilitating the degradation of ETAs, and at the same time, increasing the electrothermal performance/stability required for highly efficient treatment. In this study, we controlled the thermal signature of the MoS_2_ by harnessing MoS_2_ nanostructures with M13 phage (MNM) via the structural assembling (hydrophobic interaction) phenomena and developed a combined PANC-1 cancer cell–MNM alternating current (AC)-stimulus framework for cancer cell ablation and electrothermal therapy. A percentage decrease in the cell viability of ~23% was achieved, as well as a degradation time of 2 weeks; a stimulus length of 100 μs was also achieved. Molecular dynamics (MD) simulations revealed the assembling kinetics in integrated M13 phage–cancer cell protein systems and the structural origin of the hydrophobic interaction-enabled increase in thermal conduction. This study not only introduced an ‘ideal’ agent that avoided the limitations of ETAs but also provided a proof-of-concept application of MoS_2_-based materials in efficacious cancer therapy.

## 1. Introduction

Pancreatic cancer (PC) is the fourth leading cause of death in both men and women worldwide and has the lowest survival rate of all major organ cancers [1,2,3,4]. For instance, approximately 56,770 people were diagnosed with PC in 2019, and 45,750 deaths from this disease occurred in the United States [5,6]. Moreover, 80–85% of patients are diagnosed with advanced-stage disease, with a five-year overall survival rate of ~10% [7,8,9]. In Singapore, PC is the fourth and fifth most typical cause of cancer death in women and men, respectively [10,11]. The archetypal PC type, adenocarcinoma, is inoperable by the time patients are symptomatic [9,12]. This arises from the propensity of pancreatic tumors to spread to adjacent structures and blood vessels in the early stage [13,14]. Thus, in this work, the interest is in the development of alternative technologies that treat locally advanced diseases, which are otherwise not operable.

Cancer thermal-based therapy (TBT) is a promising candidate for achieving a minimally invasive, minimized ablation zone and a highly efficient therapeutic modality [15,16,17,18,19]. TBT operations, based on the utilization of thermal agents (TAs) to generate local hyperpyrexia, enabling the thermal elimination of tumors, have demonstrated excellent success in preclinical and clinical trials [20,21,22]. Clinical studies have disclosed that the TBT-facilitated ablation of tumors exhibits success in ~90% of patients without noticeable side effects, severe complications, or harmful changes in organ functions [19,23]. TBT is expected to have not only significant but also revolutionary clinical impact due to these promising results, as well as the recent clinical approval of the utility of metal oxide nanoparticles for the TBT in Europe [24,25]. Moreover, experiments have demonstrated enhanced pancreatic tumor ablation and photothermal therapy using polyprrole-based nanoparticles [26]. However, developing TAs with excellent thermal effects (viz., high thermal stability/performance to achieve effective therapeutic outcomes) and rapid degradability (i.e., fast degradation to address safety concerns) is extremely challenging [22,27,28].

TAs that exhibit high thermal performance under stimulations (e.g., inorganic nanomaterials) have been demonstrated in preclinical studies [29,30]. However, traditional high-performance TAs degrade with difficulty or slowly generate potential excretion problems and biosafety concerns [31,32]. Fewer safety and biocompatibility issues result when TAs with a short degradation time (viz., indocyanine green) are harnessed [33,34]. A difficulty arises from the compromise of the thermal performance, which is required for superior therapeutic efficacy, due to the rapid degradation of conventional highly degradable TAs. For instance, upon the application of a short or small number of stimulations, thermal functions disappear [35,36]. Moreover, the degradation of typical TAs further enhances upon the application of stimulations [37,38]. After incubation in aqueous solutions, two dimensional (2D) materials with metal ions that reveal a lower absorption compared to that of pristine 2D materials, indicating that the metal ions facilitate the degradation of the 2D material, have also been demonstrated in recent studies [22]. Furthermore, experiments have shown that, upon the incubation in an aqueous solution, bare nanostructures disclose a greater loss in the absorption than that of nanostructures with a polymer coating, which indicates faster degradation [39]. As a result, an impediment to fulfilling the clinical promise of TBT is represented by the balance between increasing the thermal performance and simultaneously enhancing the degradation of traditional TAs.

The molybdenum disulfide (MoS_2_) material system is a leading contender for next-generation TAs with different nanostructure types, since it is a biodegradable and biocompatible 2D material. MoS_2_ oxidizes/degrades in air and dissolves in aqueous solutions rapidly, which enhances the safety of TBT [40,41]. However, the thermal performance of traditional MoS_2_ systems is limited [42,43]. The M13 is a cylindrical, ~880 nm long, and 5–6 nm in diameter bacteriophage composed of specified proteins [44,45]. We postulate that the integration of the MoS_2_ and M13 results in MoS_2_ nanostructures with M13 (we call it MNM) that exhibit strong thermal performance, and at the same time, maintains excellent degradability. This is conceived by considering that: (i) MoS_2_ systems and M13 phages conjugate to polyethylene glycol (PEG) molecules, which maintains good degradation [46,47] and (ii) the M13 enables high thermal performance, since it carries elements to targets and assembles on tumors/cancer cells well [45].

**Figure 1:**
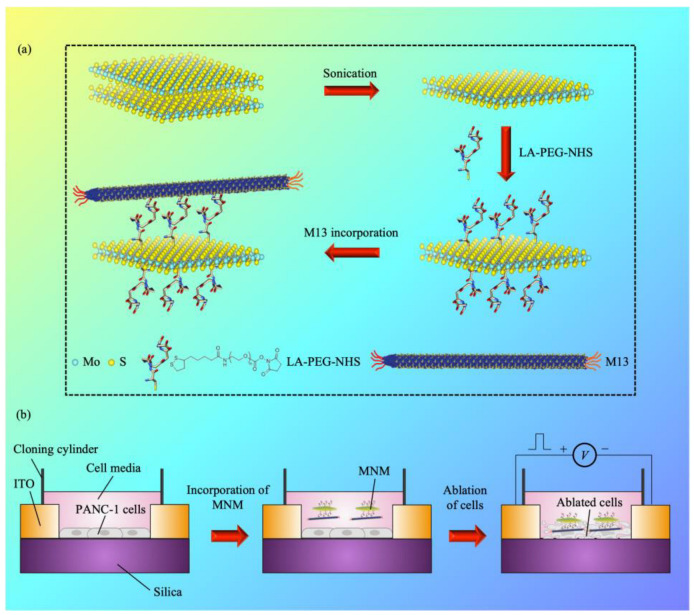
An AC-stimulus cancer cell platform based on MNM. (**a**) The bindings/reactions between the MoS_2_/M13 phage and the PEG molecules are illustrated. The schematic illustration was adapted and modified from [48]. (**b**) The MNM is incorporated in the AC-stimulus PANC-1 cancer cell system for cancer cell ablation and electrothermal therapy.

Herein we disclose that by stimulating and altering structural assembling processes, we were able to control the thermal character of the MoS_2_ by utilizing MNM, along with developing a combined alternating-current (AC) stimulus PANC-1 cancer cell MNM framework for cancer cell ablation and electrothermal therapy (Figure 1). A degradation time of 2 weeks was achieved, together with a stimulus length of 100 μs and a percentage decrease in the cell viability of ~23% upon the application of electrical stimuli. This work developed an excellent strategy to avoid the previous impasse between increasing the electrothermal performance/stability and simultaneously enhancing the degradation of electrothermal agents (ETAs).

## 2. Materials and Methods

### 2.1. Molecular Modelling

The crystal structures of the N1 and N2 domains of the M13 bacteriophage minor coat gene 3 protein (G3P) (PDB ID: 1G3P) and the extracellular domain of the programmed death-ligand 1 (PD-L1) (PDB ID: 3BIK) were retrieved from the Protein Data Bank (PDB) [49]. The oxidized Trp21 in the G3P structure was converted to Trp. Missing loop residues in G3P (residues 66–90) were modeled using the ModLoop web server [50]. Residue 18 was removed from the PD-L1 chain, as it was part of the signal peptide. The two proteins were then docked to each other using the ClusPro web server (accessed on 1 April 2022) [51]. The top five docked models were evaluated, and two were rejected, as they had G3P bound too close to the transmembrane region of the PD-L1, which would result in clashing with the cell membrane. The top-, second-, and fourth-ranked models were selected for further evaluation in molecular dynamics (MD) simulations.

The C-terminus of both protein chains was capped by an N-methyl group. Protonation states were assigned using the PDB2PQR [52] web server and then checked manually. TIP3P water molecules were utilized to solvate the systems with a minimum distance of 10 Å between the proteins and the edge of the periodic truncated octahedron solvent box. The systems were then neutralized by adding sodium ions. 

Four independent MD simulation runs were performed for each of the three selected docked models. Energy minimizations and MD simulations were performed with the PMEMD module of AMBER 18 [53] using the ff14SB force field [54]. All bonds involving hydrogen atoms were constrained by the SHAKE algorithm [55], thus allowing for a time step of 2 fs. Nonbonded interactions were truncated at 9 Å. The particle mesh Ewald method [56] was used to treat long-range electrostatic interactions under periodic boundary conditions. Energy minimization was carried out using the steepest descent algorithm for 1000 steps, followed by another 1000 steps with the conjugate gradient algorithm. The systems were then annealed gradually to 300 K over 50 ps at constant volume before equilibration at a constant pressure of 1 atm for another 50 ps. The protein non-hydrogen atoms were kept fixed with a harmonic positional restraint of 2.0 kcal mol^−1^ Å^−2^ during these minimization and equilibration steps. Subsequent unrestrained equilibration (2 ns) and production (300 ns) runs were carried out at 300 K, using a Langevin thermostat [57] with a collision frequency of 2 ps^−1^, and 1 atm, using a Berendsen barostat [58] with a pressure relaxation time of 2 ps.

### 2.2. Binding Free Energy Calculations

Binding free energies for the G3P–PD-L1 complex were calculated using the molecular mechanics/Poisson–Boltzmann surface area (MM/PBSA) method [59] implemented in AMBER 18. Two hundred equally spaced snapshot structures were extracted from the last 100 ns of each of the trajectories, and their molecular mechanical energies were calculated with the sander module. The polar contribution to the solvation free energy was calculated by the pbsa [60] program, with the solute dielectric constant set to 2 and the exterior dielectric constant set to 80, while the nonpolar contribution was estimated from the solvent-accessible surface area using the molsurf [61] program, with γ = 0.00542 kcal Å^−2^, and β = 0.92 [62]. Entropies were estimated by normal mode analysis [63] using the nmode program.

### 2.3. Electrothermal Simulations

A finite element method (FEM) was utilized in the Ansys software to analyze the thermal distribution of the integrated MNM cancer cell AC-stimulus system. Supplementary Table S1 shows the parameters utilized in the simulation. The heat conduction equation was harnessed to model the heat transfer

(1) $$\nabla \cdot k\nabla T+Q=\rho c\frac{\partial T}{\partial t}$$

where *k* is the thermal conductivity, *T* is the temperature, *Q* is the flow of Joule heat through a unit volume per unit of time, *t* is the time, *ρ* is the density, and *c* is the specific heat. The electrical stimulus with the amplitude of 1–5 V and stimulus length of 100 μs was administered to the system. The initial temperature was fixed at 37 °C (optimal temperature for mammalian cell culture).

### 2.4. Cell Lines and Cell Culture

The PANC-1 cell line was purchased from American Type Culture Collection (ATCC) and cultured in Dulbecco’s Modified Eagle’s Medium (DMEM) (Nacalai Tesque Inc., Kyoto, Japan), supplemented with 10% fetal bovine serum (FBS) (Gibco Inc., Carlsbad, CA, USA) and 1% L-glutamine (Gibco Inc., Carlsbad, CA, USA). Cells were incubated at 37 °C in the humidified incubator at an atmosphere of 5% CO_2_.

### 2.5. Escherichia coli (E. coli) and M13 Phage Propagation

The 5-alpha F’Iq competent *E. coli* (high efficiency) was purchased from New England Biolabs (NEB) as the host cell for M13 phage propagation. Overnight culture (O.C.) of *E. coli* was made with tetracycline (TET) and left on the shaker to incubate at 37 °C for 4–6 h at 90 rpm until the mixture became cloudy with an optical absorbance of 0.4 (OD600). New culture (N.C.) was prepared by incubating the O.C. in Lennox L Broth Base (LB Broth Base) at 37 °C for 4–6 h at 90 rpm. First and second precipitations were performed according to the M13 amplification protocol recommended by the manufacturer. The concentration of the M13 phage was measured using a μDrop plate (Thermo Fisher Scientific Inc., Singapore). The bacteriophage M13 15669-B1 (M13 phage) was purchased from ATCC and revived as per the phage recovery and propagation protocol (ATCC).

### 2.6. MNM Conjugation

The MoS_2_ in sterile, deionized water (DI water) was purchased from 2D Semiconductors, Inc. MoS_2_ suspension was sonicated prior to MNM conjugation to establish a homogenous mixture. The lipoic acid (LA), PEG, and N-hydroxysuccinimide ester branches (NHS) (LA-PEG-NHS) were purchased from Nanocs Inc. and reconstituted in DI water. The mixture of the MoS_2_ and LA-PEG-NHS was left to incubate on the shaker at 25 °C for 48 h under gentle shaking. The M13 phage was filtered to ensure sterility. The filtered M13 phage was then added to the mixture and left to incubate at 25 °C for 48 h under gentle shaking. Conjugated MNM was resuspended in Dulbecco’s phosphate-buffered saline (DPBS).

### 2.7. Material Characterization

Atomic force microscopy (AFM) was performed using the Bruker Dimension Icon system (Bruker Cooperation, Billerica, MA, USA) with a 1.5 μm × 1.5 μm scanning area. Samples were drop-cased on the Si substrate before AFM testing. Raw AFM data analysis was performed with the NanoScope analysis software. Raman analysis was performed using the inVia Raman microscope (Renishaw, Hoffman Estates, IL, USA) with a 532 nm excitation laser via a measured wave number in the 100–3000 cm^−1^ range. Transmission electron microscopy (TEM) imaging was performed with the field emission TEM via the FEI Talos F200 system (Thermo Scientific, Hillsboro, OR, USA), operated at a 120 kV acceleration voltage. Samples were negatively stained and drop-casted on the carbon film-coated Cu grids prior to imaging. Fourier-transform infrared (FTIR) was performed using an infrared spectroscopy (PerkinElmer, Shelton, CT, USA) after drop-casting the samples on a silicon substrate. The stability studies were performed using the MNM with 30% MN. The MNM was dispersed in the DMEM solution and maintained at 37 °C in the humidified incubator at 5% CO_2_. At predetermined time intervals, the absorbance of the MNM was measured at λ = 500–600 nm. Thermal analysis was carried out using the lock-in infrared (IR) thermography with the ELITE system (Thermo Fisher Scientific, Waltham, MA, USA) to identify hotspot locations upon the application of direct current (DC) electrical stimulus.

### 2.8. Cell Viability Studies

PANC-1 cells were plated in a 96-well plate at a seeding density of 3 × 10^3^ cells per well and left to incubate for 24 h at 37 °C in a humidified incubator at an atmosphere of 5% CO_2_. Different concentrations of the MN were added to the cells, and the cells with the MNM were allowed to incubate for 24 h at 37 °C in the humidified incubator at an atmosphere of 5% CO_2_. Cytotoxicity of the MNM was determined by the WST-1 assay performed 24 h after adding the MNM to the cells. The cells were washed once with DPBS prior to the addition of WST-1 assay.

### 2.9. Electrothermal Ablation Studies

PANC-1 cells with a density of 3 × 10^3^ cells per well were seeded in the hybrid MNM cancer cell AC-stimulus system. The system comprised two 650 nm-thick left and right ITO electrodes on the glass substrate (Latech), with a cloning cylinder secured using silicone adhesive (Sigma-Aldrich). The size of the gap between the electrodes was chosen to be 0.1 mm. To allow attachment to the glass surface, cells were cultured for 24 h. The cells were then incubated with MNM. After 24 h, electrical stimuli were applied to the system (amplitude = 1 V, 2 V, and 5 V; stimulus length = 10 μs and 100 μs; and number of stimuli = 10,000). Subsequently, the WST-1 assay was utilized 24 h after applying the stimuli to measure cell viability.

## 3. Results

### 3.1. MD Simulations

**Figure 2:**
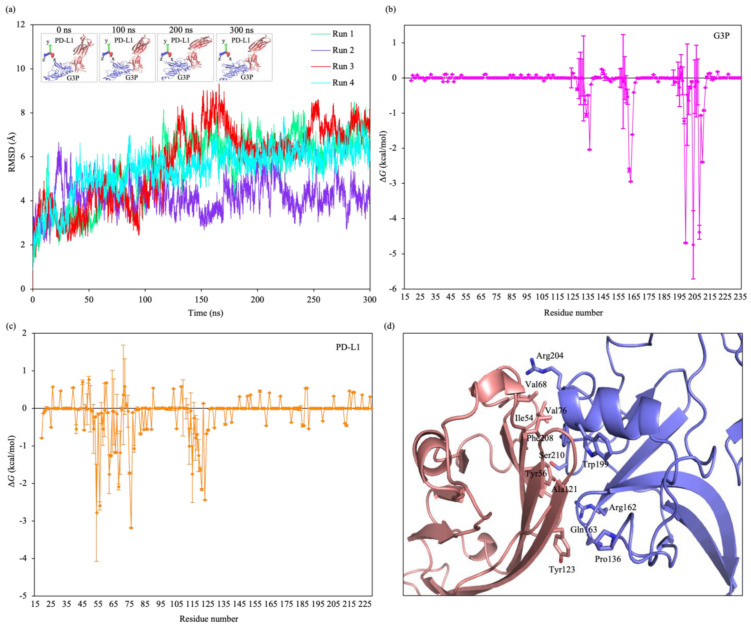
MD simulations. (**a**) Time evolution of the RMSD of backbone atoms of the M13 G3P–PD-L1 protein–protein complex. Inset, snapshots of the M13 phage protein G3P and cancer cell protein PD-L1 for different periods of run 1. (**b**,**c**) Binding free energy contributions of the (**b**) G3P and (**c**) PD-L1 residues. (**d**) Binding interface of the G3P–PD-L1 complex (purple, G3P; pink, PD-L1). The major interacting residues are shown in sticks.

Experiments have shown that overexpression of the transmembrane protein PD-L1 occurs in PC cells [64,65]. Therefore, we hypothesize that the interaction between the M13 coat protein, G3P, and the extracellular domain of the PD-L1 could account for the selectivity targeting of PC cells by MNMs. We docked the M13 minor coat protein to the PD-L1 using the ClusPro web server, and out of the top five docked models, three were selected for further evaluation in MD simulations. Based on the computed binding free energies obtained from the MD simulation, the fourth-ranked model attained from the ClusPro web server was the most stable. Its average binding free energy of 28.4 kcal/mol was lower than/comparable to that of other peptide–cancer cell protein models (Supplementary Figure S1). The time evolution of the root-mean-square displacement (RMSD) of the protein backbone atoms from the starting structure showed a gradual increase in values over the initial period (from 0 to ~120 ns) and remained stable afterwards (up to 300 ns), indicating that the system reached equilibrium (Figure 2a).

The binding interface of the complex in this model was located at the N2 domain of the G3P and the N-terminal V domain of PD-L1. To determine the residue types that are important for the stability of the interaction between the G3P and PD-L1, we performed the binding free energy decomposition based on the structures of the G3P–PD-L1 complex obtained from MD simulations. The G3P residues Pro136, Arg162, Gln163, Trp199, Arg204, Phe208, and Ser210 and PD-L1 residues Ile54, Tyr56, Val68, Val76, Ala121, and Tyr123 contributed to most of the binding free energy (Figure 2b,c). The hydrophobic nature of the binding interface between nonpolar residues on the G3P and PD-L1 caused the residues to be excluded from the polar aqueous environment and be associated with one another, allowing the proteins to interact in an end-to-end fashion. Thus, the G3P–PD-L1 interaction was stabilized by the hydrophobic core and augmented by peripheral polar interactions (Figure 2d). The breakdown of the components of the computed binding free energy (Supplementary Table S2) also indicates that the complex formation was dominated by hydrophobic interactions.

The M13 phage is also able to carry the nanomaterial to the cancer cell if the phage carries nanomaterials [66,67]. When an electrical stimulus is applied to the system, strong Joule heating occurs because the nanomaterial, e.g., MoS_2_, exhibits large electrical conductance [68,69]. As a result, the peak temperature in the cell layer (~319 K) (Supplementary Figures S2 and S3) reaches a value above the temperature required to induce cell death (315 K) [70,71], leading to excellent ablation of cancer cells.

### 3.2. Synthesis and Characterization of MNM

**Figure 3:**
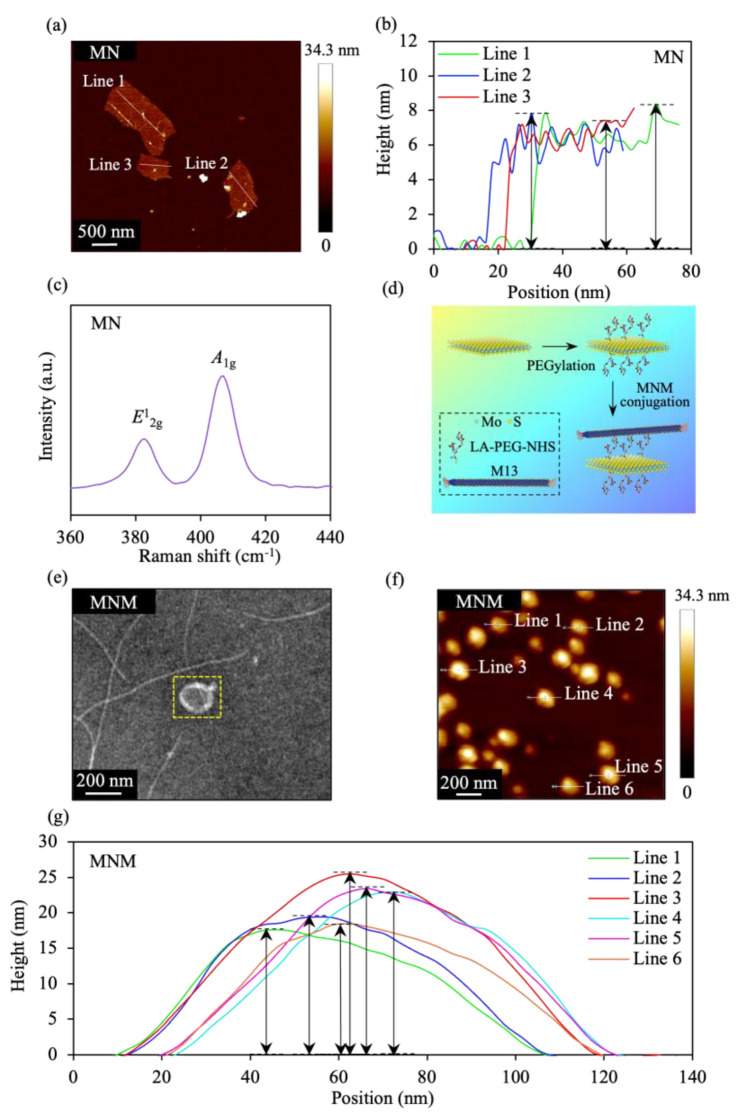
Characterization of the MN and MNM. (**a**) AFM image of MN. (**b**) Height profiles of the MN, along the white lines in (**a**). (**c**) Raman spectra of MN. (**d**) Schematic diagram of the composition and process utilized to construct MNM. (**e**) TEM image of MNM. The yellow dashed boxed area contains the head/ sheet-type structure. TEM images of the M13 phage with a diameter of ~10 nm and also a length of ~1 µm have been shown in M13-type samples harnessed by other research groups [78,79,80,84]. Our results are similar, since a similar TEM image was achieved for the sample utilized in this work. (**f**) AFM image of MNM. (**g**) Height profiles of the MNM, along the white lines in (**f**).

MoS_2_ nanostructure (MN) results from the ultrasonication of bulk MoS_2_ samples. The AFM image/cross-sectional plot discloses that the MN exhibits an average thickness of ~6.7 nm (Figure 3a,b), suggesting a stack of two triple-decker layers of MoS_2_. Moreover, Raman spectroscopy of the MN is shown in Figure 3c. The Raman spectra of the MoS_2_ is dominated by two peaks: (i) the $A_{1g}$ peak, which corresponds to the out-of-plane vibration of S atoms in opposite directions and (ii) the $E_{2g}^{1}$ peak, which is due to in-plane vibrations of two S atoms, with respect to the Mo atom [72,73]. The MN exhibits the Raman peaks $E_{2g}^{1}$ and $A_{1g}$ at ~382 and 406 cm^−1^, respectively, indicating the excellent crystal quality and structure of MN.

To enhance the stability and biocompatibility, we further modified the MN with PEG. Moreover, the M13 phage that assembles on PD-L1 cancer cell proteins was conjugated to PEG molecules to confer the cell-targeting ability to MN. The PD-L1 is a subtype of the integrin protein family that regulates angiogenesis and cancer metastasis, making it an attractive tumor cell and angiogenesis therapeutic target [74,75]. The M13/MN was conjugated to PEG molecules through amine reaction/disulfide binding (Figure 3d) [68,76,77]. The conjugation was performed by utilizing the mixture of the LA-PEG-NHS as a linker between the M13 and PEG molecules. The NHS of the LA-PEG-NHS reacted with the amine group on the M13 phage, while the LA bound to the MN via disulfide binding. Finally, the MNM was prepared, as illustrated in Figure 3d. The Fourier transform infrared (FTIR) spectroscopy was also utilized to investigate the grafting of the LA-PEG-NHS on MoS_2_ (Supplementary Figure S4). The prototypical stretching vibration of the carbonyl group in the PEG at ~1090 cm^−1^ was disclosed by the FTIR spectrum of the MNM, indicating the surface presence of PEG. The TEM image revealed that the MNM exhibited a flower-like morphology, as well as a head/sheet-type lateral size of ~200 nm (Figure 3e). For the MN, experiments have demonstrated that the diameter of the MoS_2_ nanosheet is large (~500 nm). However, for the MNM, when the PEGylation is carried out, the sonication process can partially break down these nanosheets, leading to a decrease in the diameter of MoS_2_ nanosheets. The average thickness of the MNM was ~21 nm, as shown in the AFM image/cross-sectional plot (Figure 3f,g), with an increase in the sample thickness induced by the M13 phage and PEG/polymer coating. Recent studies have shown that the M13 phage revealed a thickness of ~10 nm [78,79,80], whereas a thickness of a few nm was disclosed for the case of PEG [81,82,83].

### 3.3. MNM Thermal/Stability Signatures

**Figure 4:**
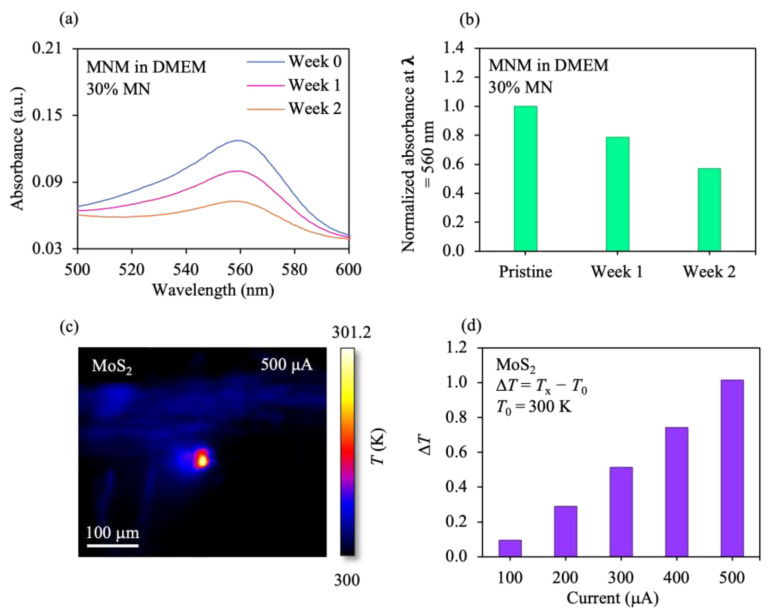
Stability and electrothermal effects of MNM. (**a**) UV-vis absorbance spectra of the MNM in the DMEM solution, with 30% MN for different weeks. The 2D material-based samples utilized by other research groups have demonstrated absorbance spectra with a decrease in the absorbance due to degradation [22,39]. A similar set of spectra were obtained for the samples used in this work, indicating that our results are similar. (**b**) Variation of the normalized absorbance at λ = 560 nm in (**a**) for the MNM in the DMEM, with 30% MN for different periods. (**c**) Thermographic map of the MoS_2_ sample upon the application of 500 μA stimulus. (**d**) Change in temperature (Δ*T*) of the sample for different stimulus currents. *T*_x_ is the temperature obtained at the targeted current.

The MNM in the DMEM solution at different times was examined. To measure the absorbance of samples, the ultraviolet visible (UV-Vis) spectrometer was utilized. For materials with a high degree of degradation, a low absorbance/normalized absorbance resulted, whereas a high absorbance/normalized absorbance value occurred for the case of the materials with a low degree of degradation [22,39]. The normalized absorbance ($A_{N}$) is given by

(2) $$A_{N}=\frac{A_{x}-A_{0}}{A_{0}}$$

where *A_x_* is the absorbance of the MNM in the DMEM at the specified week, and *A*_0_ is the absorbance of the MNM in the DMEM at week 0. At week 0, the MNM in the DMEM revealed a high normalized absorbance value (1.0), while the MNM in the DMEM solution disclosed a low normalized absorbance at week 2 (~0.56) (Figure 4a,b). Thus, a low degree of degradation was demonstrated by the MNM at week 0, and in the week-2 case, a high degree of degradation was revealed by the MNM. Experiments have also shown that most of the Mo element in MoS_2_ nanosheets were oxidized to the high valence state (Mo^VI^), suggesting the oxidation of the Mo^IV^S_2_ with a dark brown color into a colorless, water-soluble Mo^VI^-oxide species, e.g., MoO_4_^2−^ [85]. Furthermore, the stimulus current-dependent change in the peak temperature (Δ*T*) of the MoS_2_ sample was investigated (Δ*T* = temperature achieved at a specified current *T*_x_ − reference temperature *T*_0_ (300 K)). The Δ*T* increased with increasing current (Figure 4c,d), indicating that the temperature in the system can be modified with different stimulus conditions.

### 3.4. Influence of MNM on Cancer Cell Ablation

**Figure 5:**
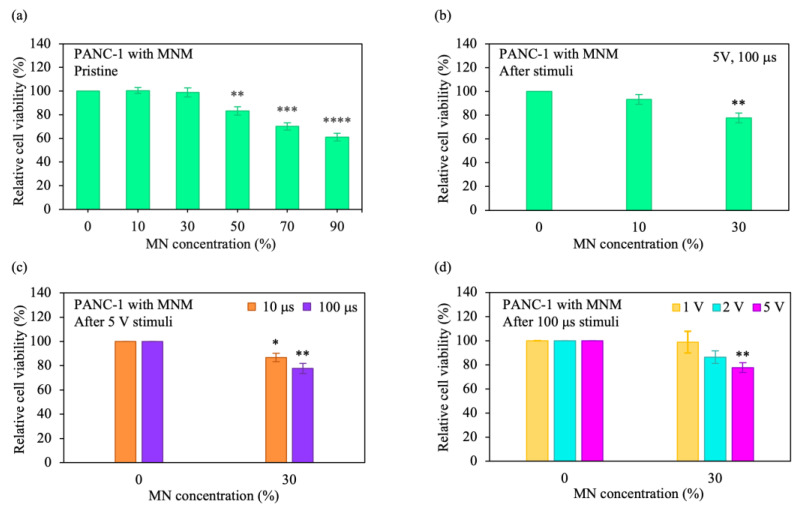
Cytotoxicity and electrothermal performance of MNM on ablating cancer cells. (**a**,**b**) Variation of relative cell viabilities of the (**a**) PANC-1 cells with MNM only and (**b**) PANC-1 cells with MNM after electrical stimuli for different MN concentrations. The cell viability was measured by the WST-1 assay, and the cells were subjected to different concentrations of MN. (**c**,**d**) Relative cell viability variations for different (**c**) stimulus lengths and (**d**) pulse amplitudes. The error bars indicate the standard error of the mean (SEM) from three independent experiments (*n* = 6). The significance values were calculated using Student’s *t*-test and are indicated as follows: * (*p* < 0.05), ** (*p* < 0.01), *** (*p* < 0.001), and **** (*p* < 0.0001). The non-significance values were unmarked.

We evaluated the influence of MN concentration on the cellular response to MNM. Experiments have demonstrated that nanosheets in cancer cells generate strong Joule heating that leads to cell death [86,87]. As the MN concentration modulates the Joule heating in the MNM, we conceived that the therapeutic influence of the MNM could be controlled by the variation in MN concentrations [28,69]. The PANC-1 cells were incubated with different MN concentrations, and the cell viability was measured to test this hypothesis. The relative cell viability is represented by

(3) $$Relative\;cell\;viability=\frac{A_{pc,\;x}-A_{pc}}{A_{pc}}\times 100\%$$

where $A_{pc,\;x}$ is the absorbance of PANC-1 cells with a targeted MN concentration, and $A_{pc}$ is the absorbance of PANC-1 cells only. PANC-1 cells with 10–30% MN disclosed a high relative cell viability (~100%) (Figure 5a). On the other hand, a low relative viability was observed for the cells incubated with 90% MN (~61%). Based on these findings, and to achieve a high initial cell viability, as well as a large conductance for strong Joule heating, samples with 10–30% MN were chosen. Upon the application of electrical stimuli, the relative viability of the cells incubated with 30% MN decreased from 100% to 77% (the cell viability decreased by 23%) (Figure 5b). Moreover, with an increase in the MN concentration from 10% to 30%, the relative cell viability after applying the stimuli decreased from 93% to 77%. Additionally, the change in stimulus conditions affected cell viability. When stimuli with an increased length were administered to the cells with 30% MN (from 10 μs to 100 μs), the relative cell viability decreased from 87% to 77%, as shown in Figure 5c. The relative viability of the cells incubated with 30% MN further decreased from 100% to 77% with increasing stimulus voltage (from 1 V to 5 V) (Figure 5d). An important part of cancer cell studies is cell morphology. The morphology of the cells with 2D materials after different treatments has been demonstrated by experiments [22,88,89]. As the samples were modified from the cells with only 2D materials to the cells treated with 2D materials and metal ions/the cells treated with 2D materials and metal ions and conditioned with the targeting agent, the cell morphology changed. The cell morphology also changed when the cells were adjusted from the cells with only 2D materials to the cells with 2D materials and metal ions and conditioned with the targeting agent, including being exposed to optical stimulation. Notably, this work demonstrated the control of thermal signatures using MNM structures, which has not been performed before, as well as developed a previously unreported combined AC-stimulus MNM cancer cell platform for cancer cell ablation and electrothermal therapy. This study also disclosed MD simulations of integrated cancer cell–M13 phage protein systems, which has not been previously carried out. These approaches allow ETAs to maintain excellent degradation, and at the same time, enhance electrothermal performance.

## 4. Discussion

Due to several requirements, applications such as cancer cell ablation and electrothermal therapy are challenging. The requirements are: (1) understanding assembly processes between the phage protein and cancer cell proteins, (2) excellent electrothermal performance, (3) good degradability, and (4) short stimulus time. Currently, the number of traditional ETAs that fulfill the requirements listed above are limited. The current state of the MNM is able to achieve most of these requirements with reasonable performance in relation to conventional ETAs, as indicated by examples disclosed in this work. The vital importance of the MNM to enable the applications is the deeper levels of insights into the hydrophobic interaction-facilitated increase in thermal conduction, which has not been demonstrated before. These results opened the door for utilizing M13 phage–cancer cell interactions in corresponding systems specifically for applications in materials science and medicine. Moreover, a percentage decrease in the cell viability of 23% in the integrated MNM cancer cell system under AC stimulation was achieved, which is ~2 times higher than the average of ~10% in current thermal-based therapy systems (Supplementary Figure S6). These results indicate that cell death in a larger population was induced for facilitating effective treatments. Additionally, the MNM exhibited a degradation time of ~2 weeks, which is 73.3% faster than the average of 7.5 weeks in existing MoS_2_-based systems in physiological media (Supplementary Figure S7), indicating that the elimination of ETAs from the systems utilized in this work occurred rapidly for enabling safe therapeutics. Furthermore, a stimulus length of 100 μs was achieved in the combined AC-stimulus MNM cancer cell platform, which is ~99.7% shorter than the average of 45 ms in state-of-the-art thermal-type therapy systems (Supplementary Figure S8). This indicates that the thermal generation is rapid for enhancing the treatment quality of patients.

## 5. Conclusions

These strong decreases in the cell viability and short stimulus length, together with good degradation, are achieved through hydrophobic interactions in integrated AC-stimulus PANC-1 cancer cell MNM systems that alter the thermal signature of MoS_2_. A different ETA concept with the potential as a unique resolution for the impasse between increasing the degradability of ETAs, and simultaneously enhancing the electrothermal performance/stability, is represented by the utilization of MNM. This study paves the way for the potential application of the MoS_2_ in combined cancer therapies.

## Data Availability

The authors declare that all data supporting the findings of this study are available within the article and the Supplementary Information. Other data are available from the corresponding authors upon reasonable request.

## Supplementary Materials

The following supporting information can be downloaded at: https://www.mdpi.com/article/10.3390/pharmaceutics15010106/s1, Table S1: Thermoelectric properties of the cell layer/nanostructure model utilized in electrothermal simulations; Table S2: Components of the computed binding free energy (kcal/mol) for the interaction of the G3P with PD-L1; Table S3: References for Figure S1; Table S4: References for Figure S6; Table S5: References for Figure S7; Table S6: References for Figure S8; Figure S1: Comparison of the averaged computed binding-free energy of the G3P–PD-L1 complex with that of current peptide-cancer cell protein models. The information of the references can be found in Table S3; Figure S2: (a) Thermal distribution of the cell-layer/nanostructure model. The MoS_2_ and PEG/M13 was inserted in the middle of the cell layer, and a square-based electrical stimulus was applied. (b) Variation of the peak temperature in the cell layer for different stimulus amplitudes; Figure S3: Thermal profile of the cell layer upon the application of 5-V stimulus; Figure S4: Fourier transform infrared (FTIR) spectra of the MNM, LA-PEG-NHS, M13 and MN; Figure S5: Variation of the relative cell viability of PANC-1 cells with MN for different MN concentrations; Figure S6: Comparison of the percentage decrease in the cell viability of integrated MNM cancer cell AC-stimulus systems with that of existing thermal-based therapy platforms. The information of the references can be found in Table S4; Figure S7: Comparison of the degradation time of the MNM with that of state-of-the-art MoS_2_-based systems in physiological media. The information of the references can be found in Table S5; Figure S8: Comparison of the stimulus length of the combined MNM AC-stimulus cancer cell platform with that of current thermal-type therapy systems. The information of the references can be found in Table S6.
