# Supplementary material for: An Efficient, Short Stimulus PANC-1 Cancer Cell Ablation and Electrothermal Therapy Driven by Hydrophobic Interactions
Source: Pharmaceutics. 2022 Dec 28;15(1):106. doi: 10.3390/pharmaceutics15010106 (PMC9867450; doi:10.3390/pharmaceutics15010106)
Supplement: Supplementary file 1 [file pharmaceutics-15-00106-s001.zip › pharmaceutics-2030333-supplementary.pdf]

Supporting Information

An efficient, short stimulus PANC-1 cancer cell ablation and electrothermal therapy driven by hydrophobic interactions

Maria Prisca Meivita,[1] Denise Lee, [1] J Shamita Naikar,[1,2] Shao Xiang Go,[1] Wey Chyi Teoh,[2,3] Yaw Sing Tan,[4] Natasa Bajalovic,[1,] Desmond K. Loke,[1,2]

[1]*Department of Science, Mathematics and Technology, Singapore University of Technology and Design, Singapore 487372, Singapore*

[2]*Office of Innovation,* [3]*Department of Radiology, Changi General Hospital, Singapore 529889, Singapore*

[4]*Bioinformatics Institute, Agency for Science, Technology and Research (A*STAR), Singapore 138671, Singapore*

Correspondence and requests for material should be addressed to N.B. (e-mail: natasa_bajalovic@sutd.edu.sg) or D.K.L (e-mail: desmond_loke@sutd.edu.sg).

Table S1. Thermoelectric properties of the cell layer/ nanostructure model utilized in electrothermal simulations.

Material	Isotropic thermal conductivity (W/mK)	Isotropic resistivity (Ω cm)
**SiO_2**	1.4	10^{16}
**ITO**	4	0.0001
**PEG/ M13**	0.2	0.1
**Cell in DMEM**	0.63	97.66
**MoS_2**	0.26	29

Table S2. Components of the computed binding free energy (kcal/mol) for the interaction of the G3P with PD-L1[a]

System	ΔE_{ele}	ΔE_{vdw}	ΔE_{PBSUR}	ΔE_{PBCAL}	ΔH_{bind}	$T\Delta S$	ΔG_{bind}
G3P-PD-L1	−2.56 ± 20.43	−80.35 ± 9.91	−10.47 ± 0.70	20.86 ± 17.50	−72.50 ± 5.78	−44.06 ± 2.67	−28.44 ± 6.89

[a]ΔE_{ele}, electrostatic potential energy; ΔE_{vdw}, van der Waals potential energy; ΔE_{PBSUR}, nonpolar contribution to solvation free energy; ΔE_{PBCAL}, polar contribution to solvation free energy; $\Delta H_{bind} = \Delta E_{ele} + \Delta E_{vdw} + \Delta E_{PBSUR} + \Delta E_{PBCAL}$, enthalpy of binding; $T\Delta S$, sum of vibrational, rotational and translational entropic contributions; $\Delta G_{bind} = \Delta H_{bind} - T\Delta S$, free energy of binding.

Table S3. References for Figure S1.

Ref No.	Reference	Type of interactions
1	Joseph, T. L.; Madhumalar, A.; Brown, C. J.; Lane, D. P.; Verma, C. S. Differential Binding of P53 and Nutlin to MDM2 and MDMX: Computational Studies. *Cell Cycle* **2010**, *9* (6), 1167–1181. https://doi.org/10.4161/cc.9.6.11067.	MDM2 and p53
2	Zhang, D.; Jia, H.; Wang, Y.; Li, W.-M.; Hou, Y.-C.; Yin, S.-W.; Wang, T. D.; He, S.-X.; Lu, S.-Y. A CD44 Specific Peptide Developed by Phage Display for Targeting Gastric Cancer. *Biotechnol Lett* **2015**, *37* (11), 2311–2320. https://doi.org/10.1007/s10529-015-1896-z.	RP-1 peptide and CD44
3	Oo, K. K.; Kamolhan, T.; Soni, A.; Thongchot, S.; Mitrpant, C.; O-charoenrat, P.; Thuwajit, C.; Thuwajit, P. Development of an Engineered Peptide Antagonist against Periostin to Overcome Doxorubicin Resistance in Breast Cancer. *BMC Cancer* **2021**, *21* (1), 65. https://doi.org/10.1186/s12885-020-07761-w.	anti-PN peptide and PN

Table S4. References for Figure S6.

Ref No.	Reference
1	Han, X.; Deng, Z.; Yang, Z.; Wang, Y.; Zhu, H.; Chen, B.; Cui, Z.; Ewing, R. C.; Shi, D. Biomarkerless Targeting and Photothermal Cancer Cell Killing by Surface-Electrically-Charged Superparamagnetic Fe_3O_4 Composite Nanoparticles. *Nanoscale* **2017**, *9* (4), 1457–1465. https://doi.org/10.1039/C6NR07161A.
2	Mantso, T.; Vasileiadis, S.; Anestopoulos, I.; Voulgaridou, G. P.; Lampri, E.; Botaitis, S.; Kontomanolis, E. N.; Simopoulos, C.; Goussetis, G.; Franco, R.; Chlichlia, K.; Pappa, A.; Panayiotidis, M. I. Hyperthermia Induces Therapeutic Effectiveness and Potentiates Adjuvant Therapy with Non-Targeted and Targeted Drugs in an in Vitro Model of Human Malignant Melanoma. *Sci Rep* **2018**, *8* (1), 10724. https://doi.org/10.1038/s41598-018-29018-0.

Table S5. References for Figure S7.

Ref No.	Reference	Type of nanostructure
1	Kurapati, R.; Muzi, L.; de Garibay, A. P. R.; Russier, J.; Voiry, D.; Vacchi, I. A.; Chhowalla, M.; Bianco, A. Enzymatic Biodegradability of Pristine and Functionalized Transition Metal Dichalcogenide MoS2 Nanosheets. *Advanced Functional Materials* **2017**, *27* (7), 1605176. https://doi.org/10.1002/adfm.201605176.	MoS_2
2	Hao, J.; Song, G.; Liu, T.; Yi, X.; Yang, K.; Cheng, L.; Liu, Z. In Vivo Long-Term Biodistribution, Excretion, and Toxicology of PEGylated Transition-Metal Dichalcogenides MS_2 (M = Mo, W, Ti) Nanosheets. *Adv Sci (Weinh)* **2016**, *4* (1), 1600160. https://doi.org/10.1002/advs.201600160.	MoS_2/PEG

Table S6. References for Figure S8.

Ref No.	Reference
1	Kim, H.; Jo, G.; Chang, J. H. Ultrasound-Assisted Photothermal Therapy and Real-Time Treatment Monitoring. *Biomed Opt Express* **2018**, *9* (9), 4472–4480. https://doi.org/10.1364/BOE.9.004472.
2	Mittelstein, D. R.; Ye, J.; Schibber, E. F.; Roychoudhury, A.; Martinez, L. T.; Fekrazad, M. H.; Ortiz, M.; Lee, P. P.; Shapiro, M. G.; Gharib, M. Selective Ablation of Cancer Cells with Low Intensity Pulsed Ultrasound. *Appl. Phys. Lett.* **2020**, *116* (1), 013701. https://doi.org/10.1063/1.5128627.

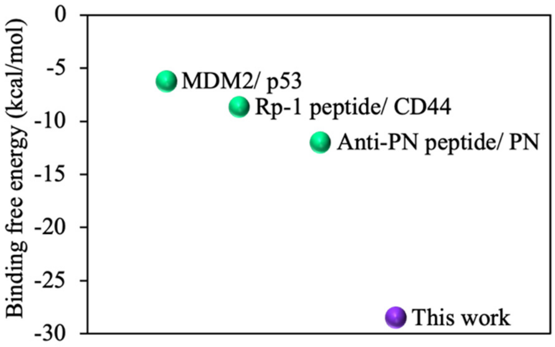

Figure S1. Comparison of the averaged computed binding-free energy of the G3P–PD-L1 complex with that of current peptide-cancer cell protein models. The information of the references can be found in Table S3.

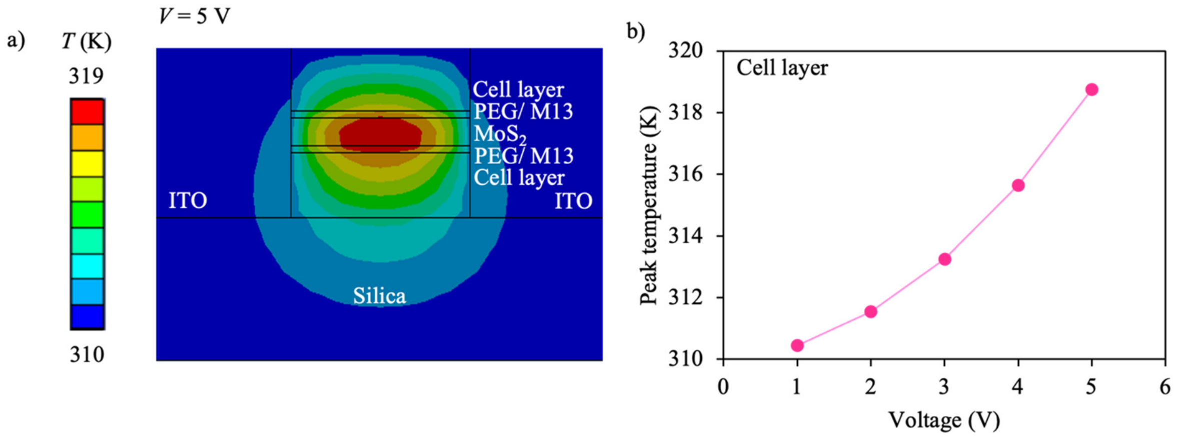

Figure S2. a) Thermal distribution of the cell-layer/ nanostructure model. The MoS_2 and PEG/ M13 was inserted in the middle of the cell layer, and a square-based electrical stimulus was applied. b) Variation of the peak temperature in the cell layer for different stimulus amplitudes.

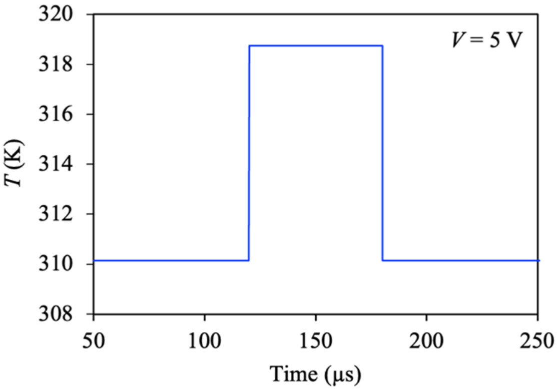

Figure S3. Thermal profile of the cell layer upon the application of 5-V stimulus.

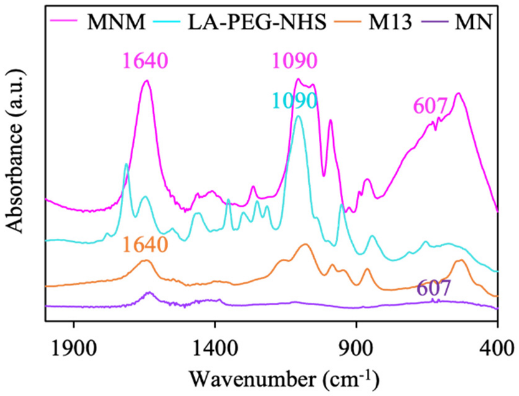

Figure S4. Fourier transform infrared (FTIR) spectra of the MNM, LA-PEG-NHS, M13 and MN.

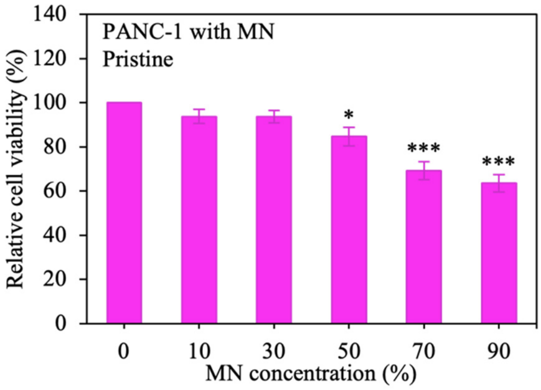

Figure S5. Variation of the relative cell viability of PANC-1 cells with MN for different MN concentrations.

Cell viability plots with a decreased behavior for the material concentration above a specified value have been shown in both the cells with 2D-based materials only and the cells with 2D-based materials and with the use of the PEG/ targeting agent harnessed by other research groups [1,2]. Our results are similar since a similar set of plots were attained for the cells with MN only/ MNM utilized in this work.

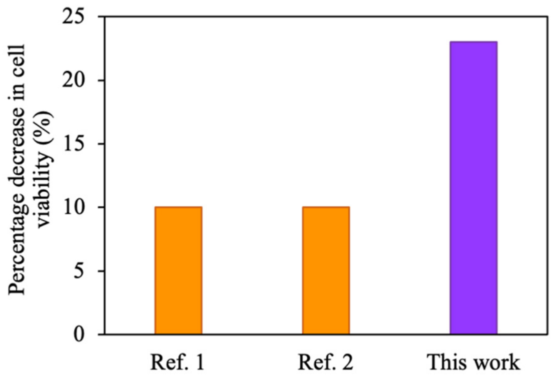

Figure S6. Comparison of the percentage decrease in the cell viability of integrated MNM cancer cell AC-stimulus systems with that of existing thermal-based therapy platforms. The information of the references can be found in Table S4.

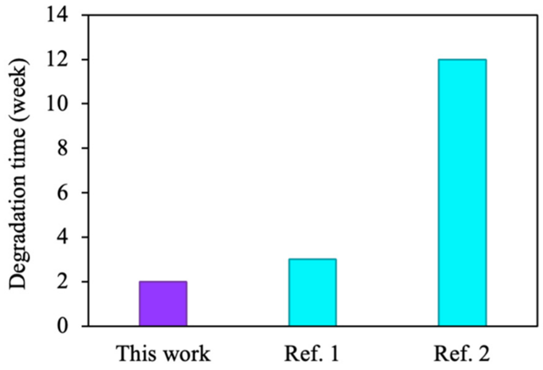

Figure S7. Comparison of the degradation time of the MNM with that of state-of-the-art MoS_2-based systems in physiological media. The information of the references can be found in Table S5.

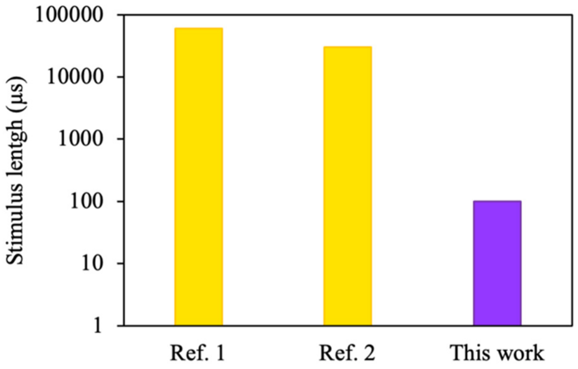

Figure S8. Comparison of the stimulus length of the combined MNM AC-stimulus cancer cell platform with that of current thermal-type therapy systems. The information of the references can be found in Table S6.

Reference

1. Hu, K.; Xie, L.; Zhang, Y.; Hanyu, M.; Yang, Z.; Nagatsu, K.; Suzuki, H.; Ouyang, J.; Ji, X.; Wei, J.; et al. Marriage of Black Phosphorus and Cu2+ as Effective Photothermal Agents for PET-Guided Combination Cancer Therapy. *Nat Commun* **2020**, *11*, 2778, doi:10.1038/s41467-020-16513-0.
2. Lu, H.; Xu, Y.; Qiao, R.; Lu, Z.; Wang, P.; Zhang, X.; Chen, A.; Zou, L.; Wang, Z. A Novel Clustered SPIO Nanoplatform with Enhanced Magnetic Resonance T2 Relaxation Rate for Micro-Tumor Detection and Photothermal Synergistic Therapy. *Nano Res.* **2020**, *13*, 2216–2225, doi:10.1007/s12274-020-2839-0.
